# A potential sensing mechanism for DNA nucleobases by optical properties of GO and *MoS*_2_ Nanopores

**DOI:** 10.1038/s41598-019-41165-6

**Published:** 2019-04-17

**Authors:** Vahid Faramarzi, Vahid Ahmadi, Bashir Fotouhi, Mostafa Abasifard

**Affiliations:** Faculty of Electrical and Computer Engineering, Tarbiat Modares University, P. O. Box 14115-194, Tehran, 1411713116 Iran

**Keywords:** Nanopores, Computational methods, Nanopores, Biological physics

## Abstract

We propose a new DNA sensing mechanism based on optical properties of graphene oxide (GO) and molybdenum disulphide (*MoS*_2_) nanopores. In this method, GO and *MoS*_2_ is utilized as quantum dot (QD) nanopore and DNA molecule translocate through the nanopore. A recently-developed hybrid quantum/classical method (HQCM) is employed which uses time-dependent density functional theory and quasi-static finite difference time domain approach. Due to good biocompatibility, stability and excitation wavelength dependent emission behavior of GO and *MoS*_2_ we use them as nanopore materials. The absorption and emission peaks wavelengths of GO and *MoS*_2_ nanopores are investigated in the presence of DNA nucleobases. The maximum sensitivity of the proposed method to DNA is achieved for the 2-nm GO nanopore. Results show that insertion of DNA nucleobases in the nanopore shifts the wavelength of the emitted light from GO or *MoS*_2_ nanopore up to 130 nm. The maximum value of the relative shift between two different nucleobases is achieved by the shift between cytosine (C) and thymine (T) nucleobases, ~111 nm for 2-nm GO nanopore. Results show that the proposed mechanism has a superior capability to be used in future DNA sequencers.

## Introduction

Rapid DNA sequencing methods are excellent tools for the growing field of personalized medicine and have been developed theoretically and experimentally^1–7^. These rapid DNA sequencers utilize any changes in the ionic or tunneling currents, surface plasmon resonances, self-aligned optical antenna and surface-enhanced Raman spectroscopy to determine type of the DNA nucleotides: adenine (A), cytosine (C), guanine (G) and thymine (T)^2,4–8^. The minimal thickness of the single-layer nanopores such as graphene is the key driving force for two-dimensional-material nanopore^2,5^. However, in order to achieve single-nucleotide resolution, there are still many other challenges such as high membrane thickness, fast DNA translocation speed, slow sensing mechanisms and noise effects^1,2,4^. In this paper, we propose and analyze a novel concept for sequencing DNA molecules by absorption and emission properties of fluorescent materials. For DNA sequencing by this new approach, we have to use molecules with excitation-dependent emission behaviours, because each DNA nucleotide has a unique absorption spectrum. Recently, semiconductor quantum dots (QDs) are proposed to be used in fluorescence emission applications, because of their advantages such as higher quantum yields (the ratio of emitted to absorbed photons from any object), controllable properties with size and shape, and resistance to photobleaching, over commercial dyes^9,10^. However, according to the Kasha’s rule^11^, the fluorescence of conventional fluorophores, such as organic dyes and semiconductor QDs, does not depend on excitation energy. This is because excited electrons are mostly relaxed to the bottom of the conduction band before the fluorescence begins, which is independent of the initial excitation photon energy. On the other hands, graphene derivatives exhibit much interesting photoluminescence (PL) properties^12,13^.

Graphene oxide GO is a functionalized few layered forms of graphene with oxygen functional groups that are attached on the basal plane. Studies show that the photoluminescence emission of GO in a polar solvent, like water, is dependent on the excitation wavelength^14,15^. The position of the fluorescence peak of GO in such polar solvent, without changing the GO sheet size, red-shifts with increasing excitation wavelength. The strong excitation wavelength dependent fluorescence in GO is originated from the red-edge effect, which results from a slowed solvation process due to an interaction between solvent dipole and fluorophore dipole^14^. Furthermore, it is shown that molybdenum disulfide (*MoS*_2_) QDs with a series of advantages, such as high quantum-yield, multicolor PL emission ranging from blue to red and good biocompatibility, have a great potential for utilizing in bio-detection applications^16,17^. Also, excitation dependent PL emission spectra in *MoS*_2_ QDs are observed and fluorescence peak position, for the uniform size of the gathered *MoS*_2_ QDs, varies under different excitation wavelength^18–20^. The aim of this study is showing a new method using optical properties of GO and *MoS*_2_ nanosheets in order to fast, label-free and accurate detection and sequencing of DNA nucleobases. The photoabsorption spectra of GO and *MoS*_2_ nanopores in the presence of DNA are calculated by employing the powerful hybrid quantum/classical method (HQCM)^21^. Next, the impact of presented DNA nucleobases at the nanopores on the photoabsorption spectra, band-gap energies, electric field enhancements and emission wavelengths of GO and *MoS*_2_ nanopores is investigated. Then, by a signal processing step, we find one frequency channel per DNA nucleobase as an excitation wavelength for each type and size of nanosheets. Regarding the excitation wavelength dependent emission properties of GO and *MoS*_2_ nanosheets, emitted light wavelengths from the GO and *MoS*_2_ nanopores are calculated and analyzed in the presence of all types of the DNA nucleobases, individually. Thus, an emission peak wavelength, as a detection signal, can be assigned for each type of DNA nucleobases. Results show a superior capability of this concept to be used in future DNA sequencers.

## The Proposed Structure and Operation Principle

**Figure 1:**
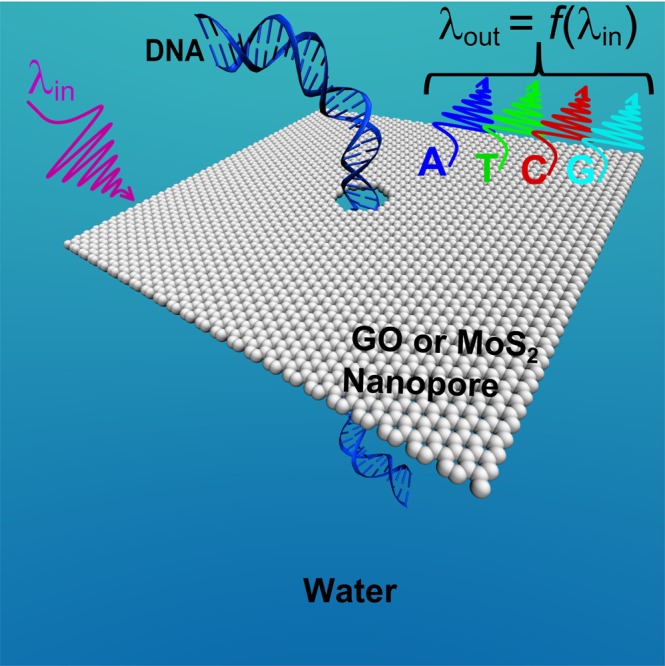
The schematic structure of our proposed DNA sequencing method based on the excitation-dependent emission property of GO or *MoS*_2_ nanopore while DNA molecule passes through the nanopore. The structure is assumed to be suspended in a polar solvent such as water. Regarding the excitation wavelength dependent behavior of GO and *MoS*_2_ materials, the emission wavelength *λ*_*out*_ would be a function of the incident light wavelength *λ*_*in*_. The blue, green, red and cyan colors represent the emission wavelength of the GO or *MoS*_2_, corresponding to the presented A, T, C and G nucleobases at the nanopore. The function (*f* ) is determined by the type of the DNA nucleobases.

The schematic structure of our proposed DNA sequencing method is presented in Fig. 1. It contains a symmetric QD nanopore while a DNA molecule is introduced in the middle of the nanopore. In the structure, the GO or *MoS*_2_ is utilized as QD nanopore, and DNA molecule translocates through the nanopore. The pore is classically created at the middle of nanosheet. In our theoretical model, the proposed GO and *MoS*_2_ structures are considered to be the square sheets with the thicknesses of 1 and 0.65 nm, respectively. Then, a pore with a diameter of 1.5 nm and the same thickness of nanosheet is made at the middle of it. Materials of the pore and surrounding medium are water and treated with optical properties of water in the classical subsystem. The nanopore membrane material and DNA molecules are assumed to be placed in an aqueous solution.

As DNA molecule has four nucleobases, we assign a unique optical signal for each type of DNA nucleobases. The influence of presented DNA nucleobase at the nanopore on the optical properties of the nanopore membrane material is investigated. For this purpose first, we need to obtain one photoabsorption spectrum for membrane nanopore + DNA nucleobase complex per each type of the presented nucleobase at the pore. The selectivity factor which is the capability of distinguishing between two different nucleobases is defined. For this purpose, we search the maximum difference between absorbance peaks in absorption spectra of the membrane nanopore + DNA nucleobases complexes. The peak wavelength of the final absorbed spectrum for which the difference between absorbance peaks of two different nucleobases is maximum is achieved. So, we obtain one frequency channel per DNA nucleobase and consider it as an excitation wavelength for the specific type and size of the membranes. In order to detect DNA nucleobases at the output of the proposed system, we look for the emission wavelength of the layer in the presence of the nucleobase at the nanopore corresponding to the achieved excitation wavelength. According to Kasha’s rule, emission wavelength should be fixed and independent of the excitation wavelength when we choose a certain dye or nanosheet. However, we have to use materials with the capability of having different emission wavelengths or excitation wavelength dependent emission properties, because we need to assign an emission wavelength for each type of the nucleobases. On the other hand, GO and *MoS*_2_ do not obey Kasha’s rule in a polar solution (such as water), and the peak emission wavelength varies by changing the excitation wavelength. Thus, taking into account the conditions mentioned above and also more significant amounts of molar absorption of DNA molecule nucleobases at the higher energies, especially above 4 eV, and biocompatibility issues we select GO and *MoS*_2_ nanopores^14,18,20^.

## Results and Discussions

**Figure 2:**
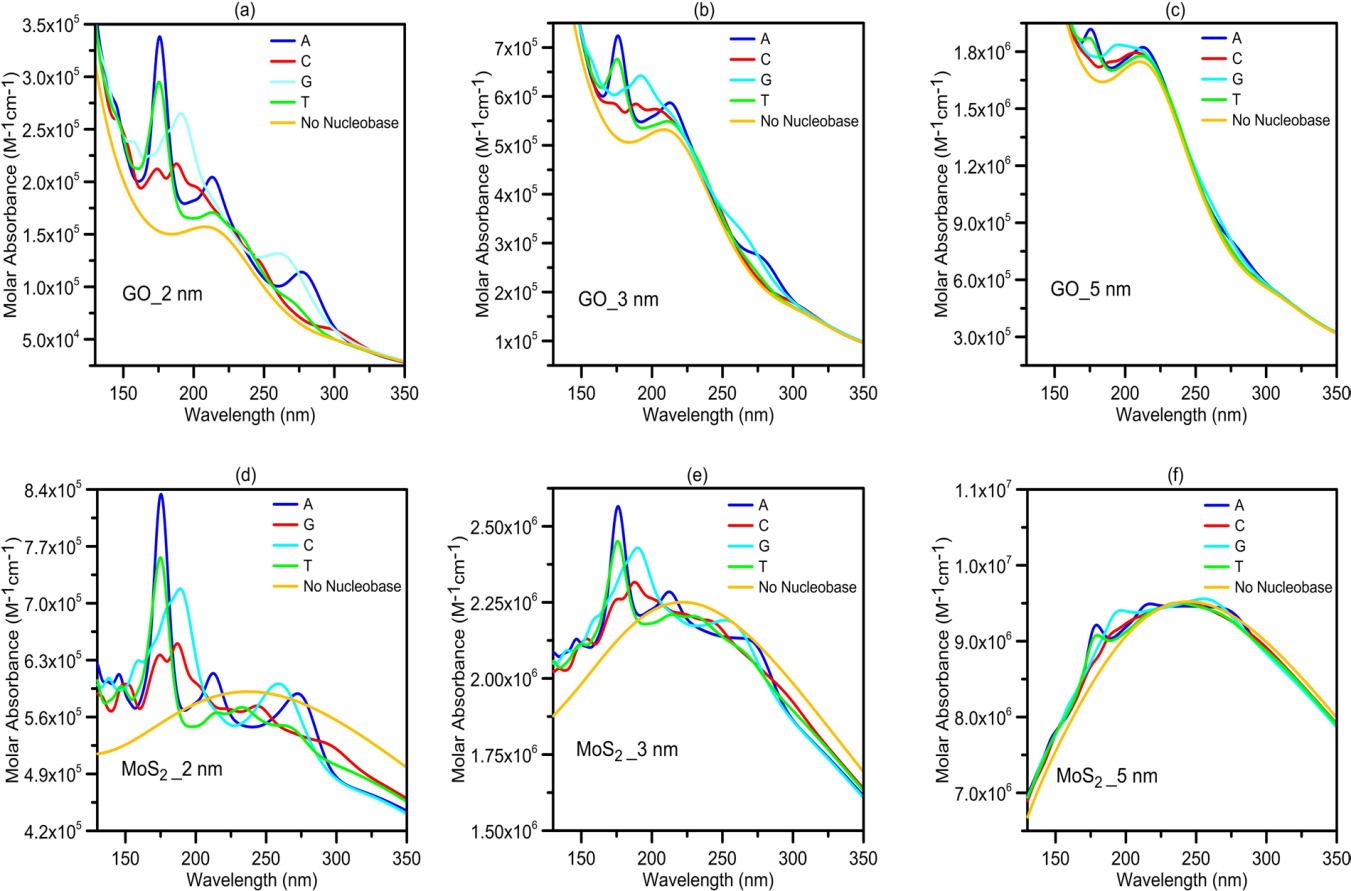
The molar absorbance for GO nanopores with the lengths of (**a**) 2, (**b**) 3 and (**c**) 5 nm, and *MoS*_2_ nanopores with the lengths of (**d**) 2, (**e**) 3 and (**f**) 5 nm in length, with and without DNA molecules. The DNA nucleobases have the most influence on the absorption spectra of GO or *MoS*_2_ nanopore with a length of 2 nm. The impacts of presented DNA molecules at nanopore reduce with increasing the length of the sheets. The thickness of GO and *MoS*_2_ nanopores are assumed to be 1 and 0.65 nm, respectively.

**Figure 3:**
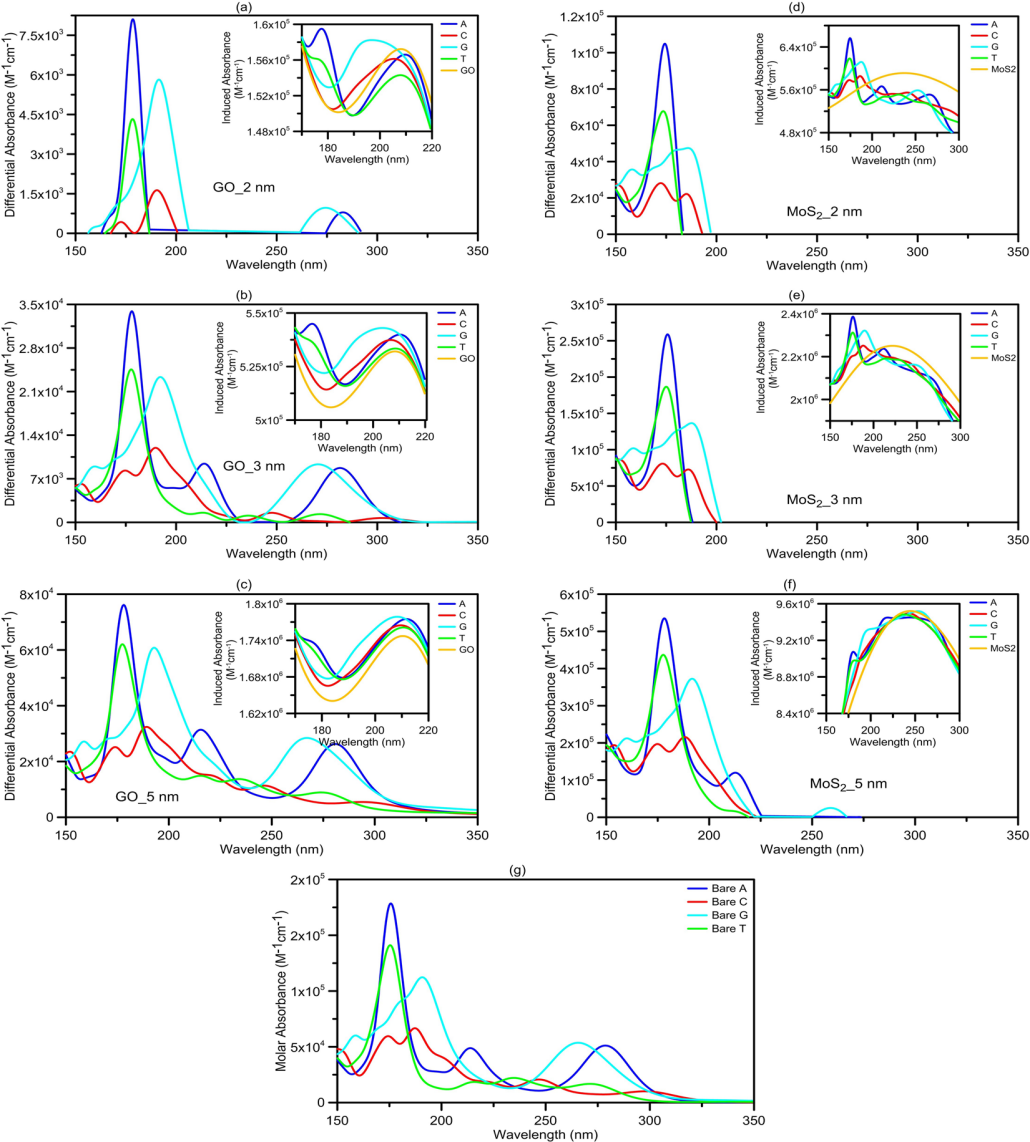
The differential absorbance of QD nanopores due to the presence of DNA molecules for GO sheets with the length of (**a**) 2, (**b**) 3 and (**c**) 5 nm, and *MoS*_2_ sheets of (**d**) 2, (**e**) 3 and (**f**) 5 nm. Different amounts of variations in differential absorbance are ascribed to different nucleobases. For lager nanosheets, a spectral line shape like bare DNA absorption spectra (**g**) can be observed in differential absorbance. Insets: the induced absorbance of differently sized GO and *MoS*_2_ nanopores due to presented DNA nucleobases at nanopore. For smaller QDs, DNA nucleobases make more peaks and shift the QD absorbance peak in the induced absorption spectra. The absorbance peaks of 5-nm sheets show no considerable shift in the presence of the DNA nucleobases. (**g**) The molar absorbance for all four bare nucleobases.

Figure 2 shows the absorption spectra for the GO and *MoS*_2_ nanopores with and without DNA nucleobases. It is assumed that the nanopore, with a diameter of 1.5 nm, to be symmetrically made in the center of GO or *MoS*_2_ nanopore and DNA molecule passes through the nanopore. The QD sheet lengths are assumed to be 2, 3 and 5 nm shown in Fig. 2(a–f), respectively. We should note that a single-stranded DNA molecule cannot pass through nanopores smaller than 1.5 nm in diameter^22^. Also, (for nanopore diameters larger than 1.5 nm) increasing the pore diameter above 1.5 nm gradually reduces the influence of the presented DNA nucleotides on the QD absorption spectra. Thus, we consider the nanopore with a diameter of 1.5 nm. For example, in Fig. 2(a–f), we can see that impact of the DNA nucleobases on the absorption spectrum of the QD nanopore is decreased by changing the sheet length from 2 to 5 nm for both GO and *MoS*_2_ nanopores. This is because the optical absorption of QDs increases with increasing the size of QDs and the impact of DNA nucleobases on the QD absorption spectrum is reduced. Generally, absorbance peaks of the QD and DNA nucleobases complex are similar to the peaks of the bare A, C, G and T nucleobases reported by Tsolakidis *et al*.^23^. For example, the dominant peaks for the A and T nucleobases are near to each other and about 7 eV (~176 nm)^23^. Similarly, in our study, and for the whole complex of QD nanopores with A or T nucleobases, the dominant introduced peaks are near to each other, at the same wavelength, that is, around 176 nm. For *MoS*_2_ nanopore and DNA molecule complex, in comparison with no nucleobase case, the absorbance increases for wavelengths smaller than 180 nm and decreases for longer wavelengths. It should be noted that in the combined system, the resonance absorbance of the DNA molecule and *MoS*_2_ nanopore are coupled, and this leads to hybridized quantum molecule-classical material states. The absorbance peak of *MoS*_2_ nanopore is strong as compared to that of the GO. On the other hand, the absorbance resonances of DNA nucleobases are significantly weaker than the absorbance peak of *MoS*_2_ nanopore, thus strong interband damping takes place for *MoS*_2_ absorbance peak when the absorbance peak of *MoS*_2_ nanopore overlaps in energy with the absorbance resonances of DNA nucleobases^24^. Because of the limited absorption intensity of DNA nucleobases at the higher wavelengths, in comparison to that of *MoS*_2_ nanopore, this interband damping effect for *MoS*_2_ nanopore can be observed in the absorption spectra of *MoS*_2_ nanopore + DNA nucleobases complexes. Also, our calculated absorbance results for the GO and *MoS*_2_ sheets are in good agreement with the experimental studies^25,26^. For more investigation of the impact of the inserted DNA nucleobases on the QD absorption spectrum, we calculate the induced absorbance of the QD due to the presence of the DNA molecule by the difference between the QD-DNA complex and the bare DNA nucleobases absorption spectra. The induced absorbance shows the net absorbance of the QD in the presence of DNA molecule. It also reveals the changes in intensity and peak position of the GO or MoS2 nanopore. Moreover, to determine the net absorbance of the system due to the presence of the DNA molecule, we calculate the difference between the induced absorbance and the absorption spectrum of the bare QD (differential absorbance). Figure 3(a–f) show the differential absorbance of GO and *MoS*_2_ nanopores for different lengths of 2, 3 and 5 nm, respectively. As can be seen in the Figure, GO nanopores show more peaks than that of the *MoS*_2_ because DNA nucleobases have more influence on the absorption spectra of the GO nanopores, due to the smaller absorbance of GO compared to that of the *MoS*_2_ nanopores. The QD nanopores with 5 nm length have higher differential absorption and show more and stronger peaks than that of the smaller QD nanopores, as shown in Fig. 3(c,f). More peaks and larger amounts of differential absorbance can be observed at lower wavelengths because of high optical absorption of DNA nucleobases in these wavelengths. The differential absorbance spectrum of larger QDs, compared to smaller ones, shows a spectral line shape like DNA absorption spectrum, as shown in Fig. 3(c,f,g). To discuss this, we show the results of the induced absorbance of QD in the presence of DNA for GO nanopores with the length of 2, 3 and 5 nm, and *MoS*_2_ nanopores of 2, 3 and 5 nm, in the insets of Fig. 3(a–f), respectively. Since optical absorption of GO and MoS2 nanopores increase when the length of the nanosheet gets larger, the effect of DNA nucleobases on the absorbance peak of the larger QD nanopores is not considerable, and the peak has no noticeable wavelength shift, as can be observed in the figures. Because DNA nucleobases have a limited optical absorption, thus for smaller QD nanopores, the induced absorbance in the presence of DNA nucleobases shows more absorbance peaks and wavelength shifting of the QD absorbance peak.

**Figure 4:**
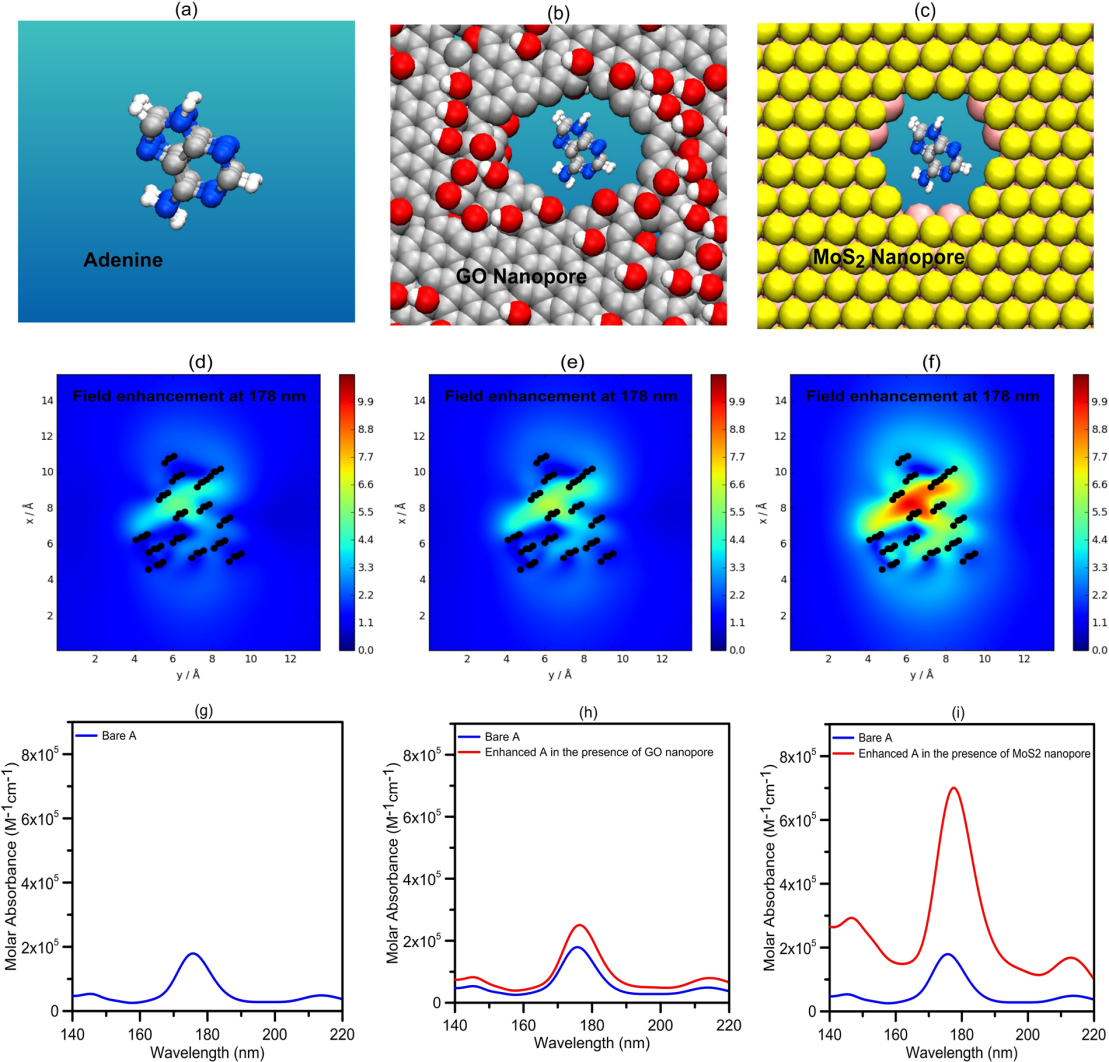
The schematic structure of (**a**) bare A nucleobases, and in the presence of (**b**) GO and (**c**) *MoS*_2_ nanopores. The electric field enhancement of (**d**) bare A nucleobases, (**e**) at the GO and (**f**) *MoS*_2_ nanopore at 178 nm. The black points show the amplified A nucleobase atoms. At the peak wavelength of 178 nm (~7 eV). The electric field of the A nucleobases at the GO and *MoS*_2_ nanopores is enhanced by a factor of 1.2 and 2, respectively. The molar absorbance of bare A nucleobases (**g**) and the enhanced absorbance of A nucleobases in the presence of (**h**) GO and (**i**) *MoS*_2_ nanopores. The enhancement factor of the A nucleobases absorbance at the presence of GO and *MoS*_2_ nanopores at the peak wavelength of 178 nm is about 1.49 and 4.1, respectively. The length of the sheets is 5 nm.

As a result, for larger nanosheets the DNA nucleobases absorption are overwhelmed by the large absorption of the nanosheets and modified absorption spectra of DNA nucleobases can be obtained by differential absorbance. Because, there is no noticeable wavelength shift in the induced absorbance for larger nanosheets, the differential absorbance of the system will be a spectrum similar to that of the original DNA nucleobases. In other words, a spectral line shape like bare DNA nucleobases absorption spectra can be obtained by calculation of differential absorbance of the system. So, differential absorbance can be useful to distinguish different DNA nucleobases presented to the nanopore. It has been shown that by considering the differential absorbance direct access to the modified dye absorbance can be achieved and a spectrum similar to that of the original dye, which is only scaled by the plasmonic enhancement factor, can be obtained^27^. Therefore, differential absorbance spectrum can provide invaluable information about the inserted DNA nucleobases at the nanopore (such as position and number of absorbance peaks) in a UV-vis absorption set-up by eliminating the background absorbance (absorbance spectrum of GO and MoS2 nanopore) of the system. The schematic structure of bare A nucleobase, and in the presence of GO and *MoS*_2_ nanopores, are shown in Fig. 4(a–c), respectively. The electric field enhancement of these configurations is calculated at the major absorbance peak wavelength of A nucleobase and shown in Fig. 4(d–f). As shown in these figures, the electric field of A nucleobase is enhanced in the presence of GO and *MoS*_2_ nanopores. The A nucleobase at the *MoS*_2_ nanopore has more field enhancement as compared to GO nanopore. Also, as shown in Fig. 4(d–f), the *MoS*_2_ nanopore has more enhancement effect on the DNA absorbance than that of the GO, because *MoS*_2_ nanopore has a stronger optical absorption than GO for a wide wavelength range from UV to near-infrared. *MoS*_2_ nanopore provides a more significant 4.1-fold enhancement in the A nucleobase absorbance, as compared to 1.49-fold enhancement with GO nanopore at the peak wavelength of ~178 nm. The enhanced absorption of DNA molecule at the GO or *MoS*_2_ nanopore verifies the results of field enhancement. Similarly, the electric field enhancement and corresponding enhanced absorption spectra of the other types of presented DNA nucleobases at GO and *MoS*_2_ nanopores, are shown in Supplementary Information Figs S1, S2 and S3. The enhanced absorption spectra have similar dominant peaks at 178, 188, 192 and 177 nm, corresponding to A, C, G and T nucleobases, respectively, compared with the bare nucleobases absorption spectra^23^ (see Fig. 4 and Supplementary Information Figs S1, S2 and S3 for more details).

**Figure 5:**
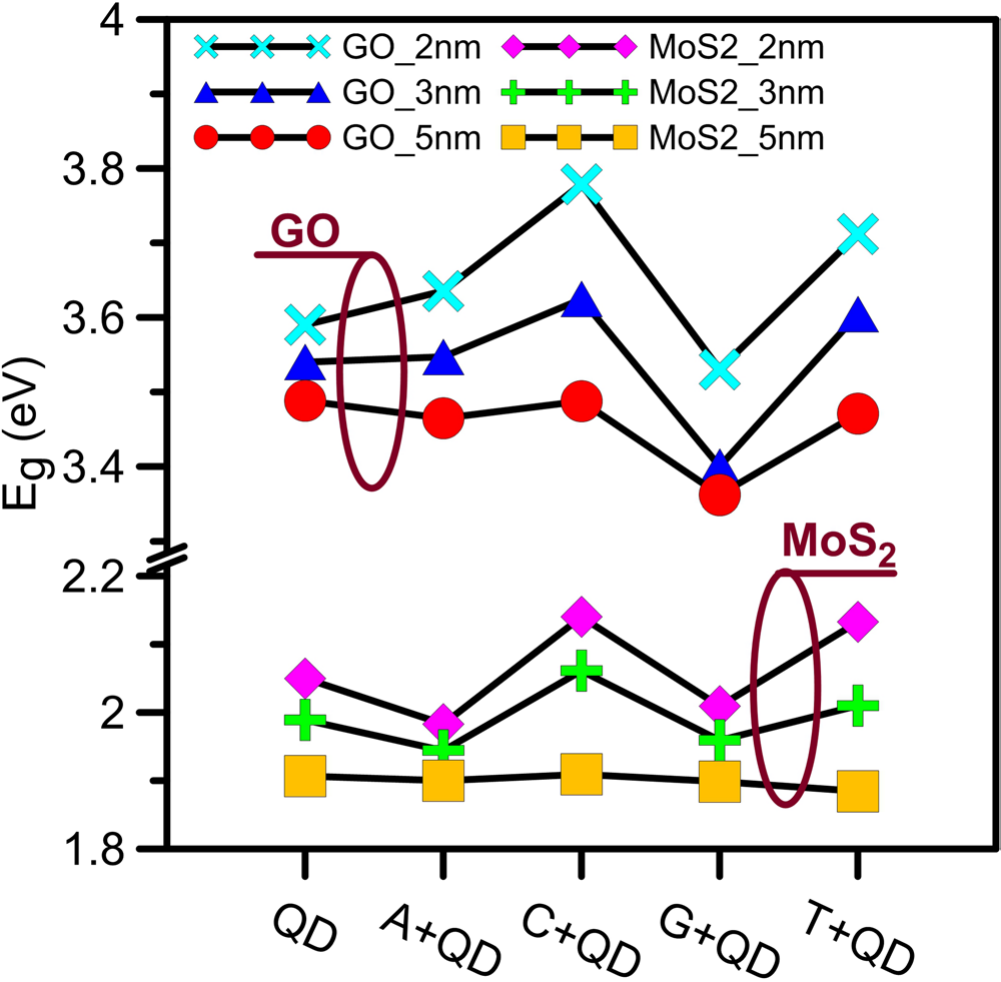
The band-gap energy of QDs in the presence of a DNA molecule. The band-gap energy of 2-nm GO sheet shows the most variations in the presence of DNA nucleobases, and ranges from ~3.53 to 3.8 eV. Also, a 5-nm *MoS*_2_ sheet has the minimum variations of the band-gap energy.

To investigate the influence of the inserted DNA molecule on the band-gap energy of QD nanopore, we calculate the band-gap energy of the GO or *MoS*_2_ nanopore and DNA molecule complex using Tauc plots^28^. As shown in Fig. 5, the band-gap energies of 5-nm GO and *MoS*_2_ sheet are ~3.5 eV and ~1.89 eV, respectively, which are in good agreement with the results presented by Mathkar *et al*.^29^ and Arul *et al*.^30^. As it is indicated in Fig. 5, the band-gap energy ranges from ~3.53 to 3.8 eV for a 2-nm GO sheet and from ~1.98 to 2.14 eV for a 2-nm *MoS*_2_ sheet in the presence of DNA molecule.

Applicability of the proposed method for DNA sequencing is influenced by a combination of the spectral shape of the input light and QD size. Here, we consider QD nanopores with lengths of 2, 3 and 5 nm, because increasing the size of the QDs reduces the average sensitivity of QDs to the presence of DNA, as shown in Fig. 2. Next, for DNA sequencing, we define figure-of-merit (FOM) given by

1 $$\begin{array}{l}FOM=\prod _{\begin{array}{c}i,j=1,2,3,4\\ i\ne j\\ i < j\end{array}}|\frac{{\lambda }_{i}-{\lambda }_{j}}{{\lambda }_{j}}|\end{array}$$

**Figure 6:**
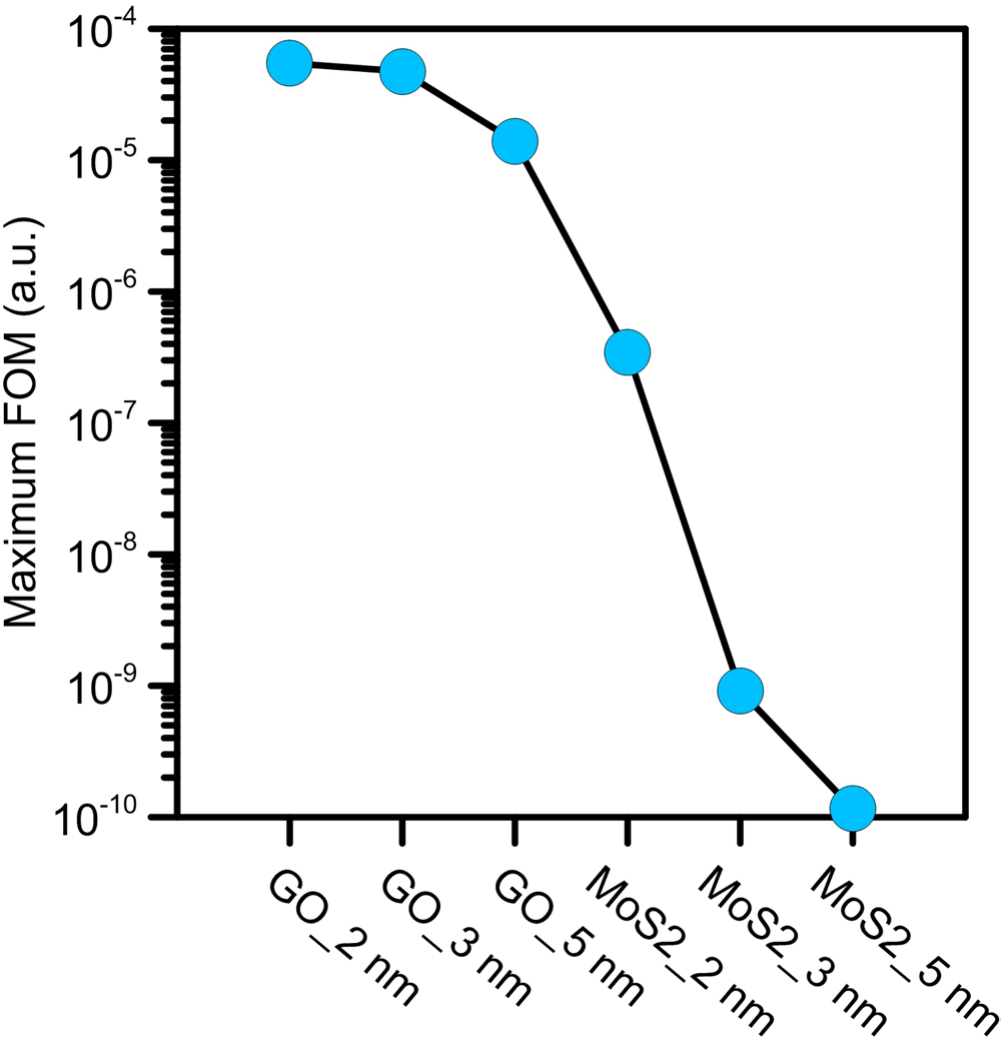
The maximum achieved FOM for GO and *MoS*_2_ nanopores. The maximum FOM is obtained under conditions in which the center frequencies are 2.88, 2.68 and 2.66 eV, for 2, 3 and 5-nm GO sheets, and 3.95, 6.14 and 6.71 eV, for 2, 3 and 5-nm *MoS*_2_ sheets, respectively.

**Figure 7:**
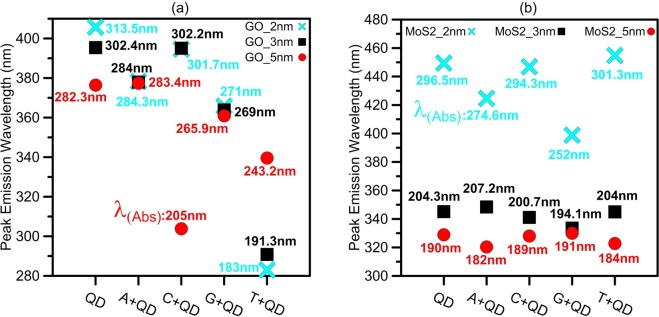
Peak emission wavelengths of all (**a**) GO and (**b**) *MoS*_2_ nanopores with and without DNA nucleobases corresponding to the center frequency and spectral width of the best achieved FOM for each type of QD nanopore. The peak absorption wavelength is labeled for each peak emission wavelength in the figures.

It enables us to distinguish between the nucleobases absorption characteristic in DNA sequencing. Here, *i* and *j* stand for possible types of DNA nucleotides: A, C, G and T. The *λ*_*t*_ is defined as the peak wavelength of the final absorption spectrum from the QD nanopore while the influence of the presented type i nucleobase to the nanopore is considered. To calculate FOM, we apply a specific function to the absorption spectrum of each type of the QD nanopore. The desired function is defined as a Gaussian function with a central frequency and a spectral width of *ω*_*c*_ and *σ*_*c*_, respectively. *ω*_*c*_ and *σ*_*c*_ are calculated to achieve the maximum value of FOM for each type of the QD. Figure 6 shows the maximum FOMs obtained for the corresponding Gaussian functions with central frequency and spectral width changed from 3 to 8 eV and 0.1 to 1.5 eV, respectively. As the Fig. 6 shows, the best FOM corresponds to 2-nm GO sheet with the Gaussian function of *ω*_*c*_ = 2.88 eV and *σ*_*c*_ = 1.39 eV. Moreover, for 2-nm *MoS*_2_ sheet, the best FOM is achieved for *ω*_*c*_ = 3.95 eV and *σ*_*c*_ = 1.38 eV. Then, we search for the peak wavelengths (*λ*_*i*_) and peak widths of the absorbed light by the QD nanopore influenced by presented DNA nucleobases, corresponding to the best value of achieved FOM. The calculated peak wavelengths of the absorbed light from GO and *MoS*_2_ nanopores corresponding to the best value of achieved FOM, with and without DNA nucleobases are demonstrated in Fig. 7.

We consider each peak wavelength of the absorbed light as an excitation wavelength for GO or *MoS*_2_ nanopore. Next, we calculate peak emission wavelength of the structures in the presence of each nucleobase. For this purpose, we find the PL peak positions based on the excitation wavelength dependent emission property of GO and *MoS*_2_ nanopores. It is shown that when the GO sheet is suspended in a polar solvent, the emission peak of GO is red shifted from 440 to 580 nm by increasing the excitation wavelength from 350 to 500 nm in water at room temperature. This results in creating a linear relationship, with a constant slope of ~1, between the emission and excitation wavelengths up to ~460 nm^14^. Also, *MoS*_2_ QDs, with and without considering the solvent effect, show variable PL emission under different excitation wavelengths and PL peak position is red shifted for the excitation wavelength within 405–552 nm^18,20^. Then, we calculate the peak emission wavelengths corresponding to the absorbed light wavelengths.

Figure 7 shows the calculated peak wavelengths of the light absorbed and emitted from all three sizes of the GO or *MoS*_2_ nanopores and DNA nucleobases complexes. For example, 3-nm GO sheet has the emission peaks centered at 337.9, 395, 363.8 and 290.8 nm corresponding to the absorbed light peaks centered at 284, 302, 269 and 191.3 nm, respectively.

**Figure 8:**
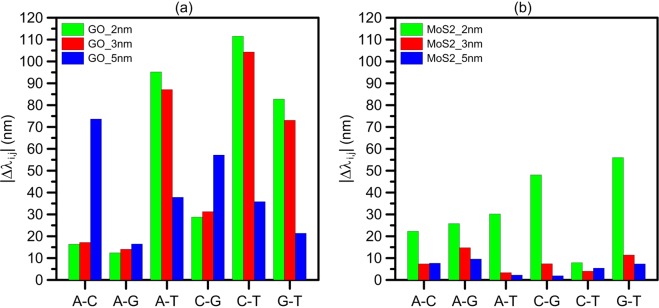
The relative shift of the main peak of the output emitted light from the QD nanopores between different nucleobases are shown for 2–5 nm (**a**) GO, and (**b**) *MoS*_2_ sheets. 2-nm GO sheet has the most sensitive emitted light to the type of DNA nucleobases. In all GO nanopores A and G show the minimum relative shifts, and the shift between C and G in 5-nm *MoS*_2_ sheet is the minimum value of the relative shift. 2-nm GO sheet has the most sensitive emitted light to the type of DNA nucleobases, and the maximum relative shift of ~112 nm is obtained by shift between C and T. In all GO nanopores, A and G show the minimum relative shifts of 12.4–16.5 nm, and a 2-nm shift between C and G in 5-nm *MoS*_2_ sheet is the minimum value of the relative shift.

To demonstrate the capabilities of the proposed structures for DNA sequencing, a relative shift of emitted light wavelength from GO and *MoS*_2_ nanopores between two different nucleobases is calculated. Figure 8 shows the relative shift of the output light wavelength of GO and *MoS*_2_ nanopores between two different nucleobases. The possible cases are A–C, A–G, A–T, C–G, C–T, and G–T. For GO (*MoS*_2_) nanopore, the maximum value of relative shift is obtained by the shift between C and T, Δ*λ*_(*C*,*T*)_ = 111.54 nm, (G and T, Δ*λ*_(*G*,*T*)_ = 56 nm,), while 2-nm GO(2-nm *MoS*_2_) sheet is used as QD nanopore. The shift between C and G in 5-nm *MoS*_2_ sheet is the minimum value of the relative shift. Here, we define the average sensitivity as

2 $$\begin{array}{l}{S}_{avg}=\frac{1}{4}\sum _{j}\sum _{i}\frac{|{\lambda }_{max,j}-{\lambda }_{max,i}|}{{\lambda }_{max,j}},\,\,\,i,j=A,C,G,T.\end{array}$$

where *λ*_*max*_ is the peak emission wavelength of GO or MoS2 nanopore, i and j are types of the DNA nucleobases. The maximum sensitivity of our proposed method to the presented DNA nucleobases is ~52.2%, which corresponds to 2-nm GO nanopore. This value is higher than the maximum sensitivities for the plasmonic- based DNA sequencing studies with values of 19% and 38% reported in^5,6^. Also, the maximum sensitivity for Surface-enhanced Raman based method is about 34.22%^7^.

It should be noted that in the field of nanopore DNA sequencing, in most cases, the main purpose of modeling and simulation is to bring a new idea or class of DNA-sequencing mechanism to this field. Nevertheless, practical parameters and challenges such as pore size, salt solution, translocation dynamics, and nucleobases stick on the pore, effects of noise signal from neighboring nucleobases, contaminations and defects are still present and unknown. From the practical point of view, a study by Yanagi showed that smaller nanopores with a diameter of 1 to 2 nm could be fabricated using dielectric breakdown. This method can generate nanopores with diameters of sub-1 nm in a 10-nm-thick Si3N4 membrane with good stability^31^. To prevent the nucleobases from sticking to the pore and thus the accumulation of DNA molecules inside the nanopore in the real experiment the pore can be passivated with a protein layer, insulating layer or specific atoms, resulting in an enhancement in the accuracy of the optical measurements and noise level reduction. According to studies from several groups, passivating the surface and the sidewall of a nanopore device can be done using bovine serum albumin (BSA)^32^, photo-definable PDMS (P-PDMS)^33^ and silicon atoms^34^ which result in preventing aggregation of DNA inside the pore, but otherwise do not significantly affect DNA translocation.

Generally, using the proposed method for sequencing DNA molecules has some advantages over the previous methods such as ionic or tunnelling currents, Raman spectroscopy and surface plasmon resonances^1–8^. The nanosecond-order lifetime of the method is both advantage and disadvantage for DNA sequencing, simultaneously. This is because DNA translocation time is short, but emission lifetime is large. This larger lifetime can be used to the simple tracking of the sensing signal. Also, DNA amplification can be utilized to give enough time for the emission mechanism to be complete. Moreover, because of size-dependent adjustability of the optical properties of QDs, and practical viability of nanometer-sized QDs, the proposed mechanism seems to be more reliable than ionic and tunnelling currents, surface plasmons and Raman spectroscopy. This concept shows more significant amounts of wavelength shifts due to presentation of DNA nucleobases. Hence, the method is more sensitive and selective compared to ionic, tunnelling, plasmonic and Raman-based mechanisms for DNA sequencing^2–8^. Also, due to higher selectivity, the suggested method can determine the type of the presented DNA nucleobases to the nanopore.

## Methods

The Hybrid Quantum/Classical Method (HQCM) has been developed for computing electronic and optical properties of semiconductors and metallic nanostructures using the real and imaginary parts of the refractive index^21,24,35,36^. This method shows acceptable agreement between modeling and experimental data^24,35^. In this method, the calculations are divided into two parts: the quantum subsystem, which is propagated using Time-Dependent Density Functional Theory (TDDFT) scheme, and classical subsystem that is treated using Quasistatic Finite-Difference Time-Domain method (QSFDTD). This method employs dipole approximation with neglecting the magnetic field^35,37^. The subsystems share a common electrostatic potential, while they are propagated separately in their own real space grids. In the Time-propagation TDDFT part of the calculation the electrostatic potential is known as the Hartree potential, ∇^2^*V*^*qm*^(**r**, *t*) = −4*πρ*^*qm*^(**r**,*t*), and in the QSFDTD method the electrostatic potential is solved from the Poisson equation as well ∇^2^*V*^*cl*^(**r**, *t*) = −4*πρ*^*cl*^(**r**,*t*). The hybrid scheme is created by replacing in both schemes the electrostatic potential by a common potential as ∇^2^*V*^*tot*^(**r**, *t*) = −4*π*[*ρ*^*cl*^(**r**, *t*) + *ρ*^*qm*^(**r**, *t*)]^24^. Then, this total potential is used in the Kohn-Sham density functional theory scheme (KS-DFT), and the electronic structure is solved for the ground state and excited state electron density. Finally, using the electron density and solving the time dependent Schrödinger equation the photoabsorption spectrum is extracted from the time-propagation simulations.

In our study, GO and *MoS*_2_ nanopores are treated with classical subsystems, and DNA molecule is treated with the quantum subsystem. Since the membranes are thicker than the distance between two adjacent nucleobases (0.34 nm) we use amplified DNA to make sure nanopore is filled with just one type of nucleobase. So, we use four-fold amplified nucleobases (1 nm) for each specific type of DNA nucleobases, equal to the highest membrane thickness (GO membrane).

For classical subsystem modeling, permittivity is modeled as a linear combination of Lorentz oscillators, as demonstrated in

3 $$\begin{array}{l}\varepsilon (\omega )={\varepsilon }_{Re}(\omega )+i{\varepsilon }_{Im}(\omega )={\varepsilon }_{\infty }+{\varepsilon }_{0}\,\sum _{j}\frac{{\beta }_{j}}{{{\omega }_{j}}^{2}-i\omega {\alpha }_{j}-{\omega }^{2}}\end{array}$$

here, *β*_*j*_, *ω*_*j*_ and *α*_*j*_ are parameters to fit desired model to the experimental permittivities. In Eq. 3, the frequency *ω* is presented in *eV*, *ε*_*Re*_ and *ε*_*Im*_ are real and imaginary parts of permittivity, respectively^24^. To find fitting parameters we search minimum value of

4 $$\begin{array}{l}\int \,\sqrt{A{({\varepsilon }_{Re}(\omega )-{\varepsilon }_{1}(\omega ))}^{2}+B({\varepsilon }_{Im}(\omega )-{\varepsilon }_{2}{(\omega )}^{2}}d\omega \end{array}$$

where *ε*_1_ and *ε*_2_ are real and imaginary parts of experimental permittivity, respectively, *A* and *B* are constant parameters which can be set to achieve the optimal fitting. The experimental permittivities for GO and *MoS*_2_ have already been reported in literature^38,39^.

Note that the introduced single-stranded DNA molecule to the QD nanopore is assumed to be single-type (only A, C, G or T) and DNA molecule length is considered to be almost equal to the diameter of the GO membrane. In the HQCM calculations, we use 1 and 0.25 Å… real-space grids for the classical and quantum subsystems, respectively, and the distance between the atoms and the grid borders is 0.4 nm. In these calculations, the time evolution is followed for 20 fs with 10 attosecond time steps, and the spectra are convoluted with Gaussian FWHM of 0.35 eV. For quantum subsystem, atomic coordinates of the relaxed DNA molecules are presented to the center of the nanopore. The main parameters for relaxation of DNA molecules and ground state calculations are basis-set = ‘dzp’, exchange-correlation functional = ‘LDA’, MeshCutoff = 200 Ry and QuasiNewton minimizer. The optimization algorithm runs until all atomic forces are below 0.05 eV per Angstrom. It should be noted that more accurate results will be obtained if DNA nucleobases are relaxed with GGA functionals. However, we have compared the calculations results of LDA with those of GGA (unpublished results). We find that there is no considerable difference between the LDA and GGA calculations. Therefore, in this study, regarding the computational time and cost of GGA functionals, the LDA functionals have been utilized. The HQCM is accurate under the condition in which characteristics dimensions of the system is smaller than the input light wavelength. For example, if the structure size is about 50 nm, the results are valid up to 6 eV^21^. Previous researches show that DNA is naturally a fluorescent molecule^40^. Thus, the excitation light is absorbed and also emitted by both GO or *MoS*_2_ nanopore and DNA molecule composition, as a complex molecule. Hence, to study molecule absorbance and emission, we consider the whole complex of the GO or *MoS*_2_ nanopore and DNA molecule. For the HQCM calculations we use GPAW codes^36,41,42^. The absorbance spectrum of the whole complex of the QD and DNA is calculated by

5 $$\begin{array}{l}Molar\,Absorbance\,(\omega )=\frac{2{\pi }^{2}N}{{10}^{3}ln10}(\frac{{e}^{2}}{mc})S(\omega )\,\,\,({M}^{-1}c{m}^{-1})\end{array}$$

where N, is Avogadro’s number and c is the velocity of light^43^. In Eq. 5 *S* is dipole strength function, along the direction parallel to the base plane of QD sheets and DNA molecule, which is numerically extracted by HQCM codes.

## Conclusion

We presented a novel method based on optical properties of GO and *MoS*_2_ QDs for sequencing DNA molecules. The mechanism combined with the nanopore-based DNA translocation is suggested and analyzed for sequencing DNA molecules. The recently developed HQCM which employs TDDFT and QSFDTD calculations are utilized to investigate impacts of DNA nucleobases on the absorption spectrum of the QD nanopores. Due to biocompatibility, stability, large band-gap energy and importantly excitation dependent PL properties, the GO and *MoS*_2_ nanopores are selected as nanopore materials. Effect of presented DNA nucleobases at the nanopores on the different parameters of the proposed method such as absorbance spectra, electric field enhancement, band-gap energies and emission peaks wavelengths of GO and *MoS*_2_ nanopores, are studied. The effect of different GO and *MoS*_2_ nanopore sizes on the proposed method is investigated. The best condition for the proposed DNA sequencing application is obtained while the GO nanopore length is 2 nm, and central frequency and spectral width of the applied Gaussian function is 2.88 and 1.39 eV, respectively. Results show that the presentation of each type of DNA nucleobases in the GO or *MoS*_2_ nanopore can change the wavelength shift of the emitted light between 1 to 130 nm. The large amounts of the wavelength shifts due to presented DNA to the nanopore, lead to higher sensitivity and selectivity compared with ionic, tunnelling, plasmonic and Raman-based methods in DNA sequencing. The results show that the proposed concept can clearly determine the type of unknown DNA nucleobases. Our study proves that the proposed method can be effectively used to sequence DNA molecules. Proposed mechanism and the results shed light on a new class of DNA sequencers for future personalized medicine.

## Supplementary information

A potential sensing mechanism for DNA nucleobases by optical properties of GO and MoS2 Nanopores
