# Supplementary material for: A potential sensing mechanism for DNA nucleobases by optical properties of GO and MoS2 Nanopores
Source: Sci Rep. 2019 Apr 17;9:6230. doi: 10.1038/s41598-019-41165-6 (PMC6470134; doi:10.1038/s41598-019-41165-6)
Supplement: Supplementary file 1 — A potential sensing mechanism for DNA nucleobases by optical properties of GO and MoS2 Nanopores [file 41598_2019_41165_MOESM1_ESM.doc]

**Supporting Information**

**A potential sensing mechanism for DNA nucleobases by optical properties of GO and MoS_2_ Nanopores**

Vahid Faramarzi, Vahid Ahmadi*, Bashir Fotouhi, and Mostafa Abasifard

School of Electrical and Computer Engineering, Tarbiat Modares University, Tehran-Iran.

*v_ahmadi@modares.ac.ir.

**Electric field enhancement and enhanced absorption spectra of presented C, G and T nucleobases at GO and MoS_2_ nanopores**

As mentioned previously in the main text, the GO and MoS_2_ nanopores have an enhancement effect on the DNA nucleobases absorption spectra. The enhanced absorption of DNA molecule at the GO/MoS_2_ nanopore verifies the results of field enhancement. This electric field enhancement is calculated at the major absorbance peak wavelengths of DNA nucleobases. For example, as shown in Figures S1-S3, the enhancement factor of electric field for presented C, G and T nucleobases at the 5 nm GO nanopore, is about 1.22, 1.25 and 1.2, respectively. In the case of MoS_2_ nanopore, this enhancement factor is about 2.07, 2.11 and 2.06 for C, G and T nucleobases, respectively. Due to larger absorbance of the MoS_2_ nanosheet than that of the GO nanosheet, the presented DNA nucleobases at the MoS_2_ nanopore have more enhanced electric filed, and thus more enhanced absorbance, as compared to GO nanopore.

| **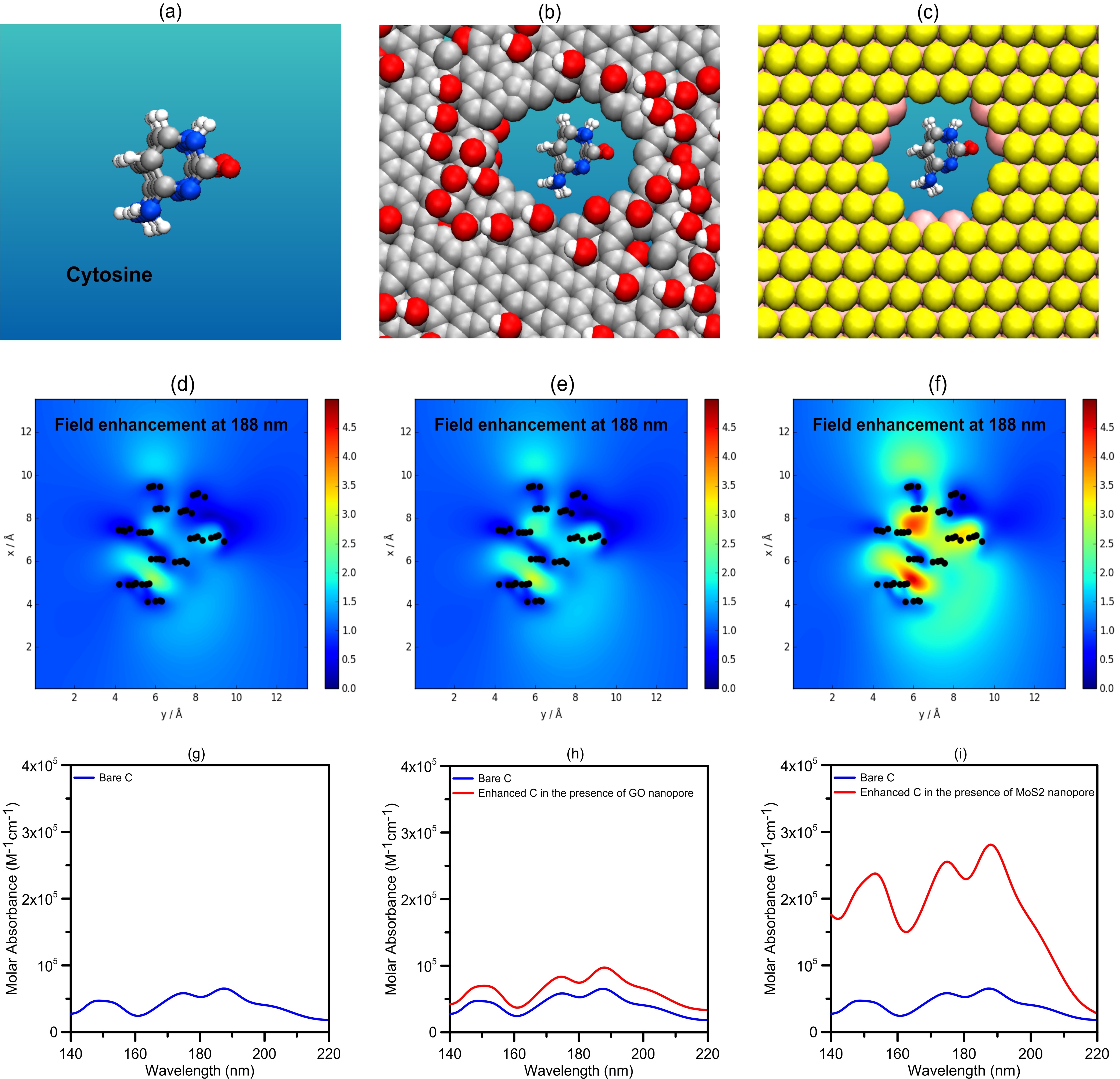** |
| --- |

**Figure S1.** The schematic structure of (a) bare C nucleobases, and in the presence of (b) GO and (c) MoS_2_ nanopores. The electric field enhancement of (d) bare C nucleobases, (e) at the GO and (f) MoS_2_ nanopore at 188 nm. The black points show the amplified C nucleobase atoms. At the peak wavelength of 188 nm (6.59 eV). The electric field of the C nucleobases at the GO and MoS_2_ nanopores is enhanced by a factor of 1.22 and 2.07, respectively. The molar absorbance of bare C nucleobases (g) and the enhanced absorbance of C nucleobases in the presence of (h) GO and (i) MoS_2_ nanopores. The enhancement factor of the C nucleobases absorbance at the presence of GO and MoS_2_ nanopores at the peak wavelength of 188 nm is about 1.49 and 4.31, respectively. The length of sheets is 5 nm.

| **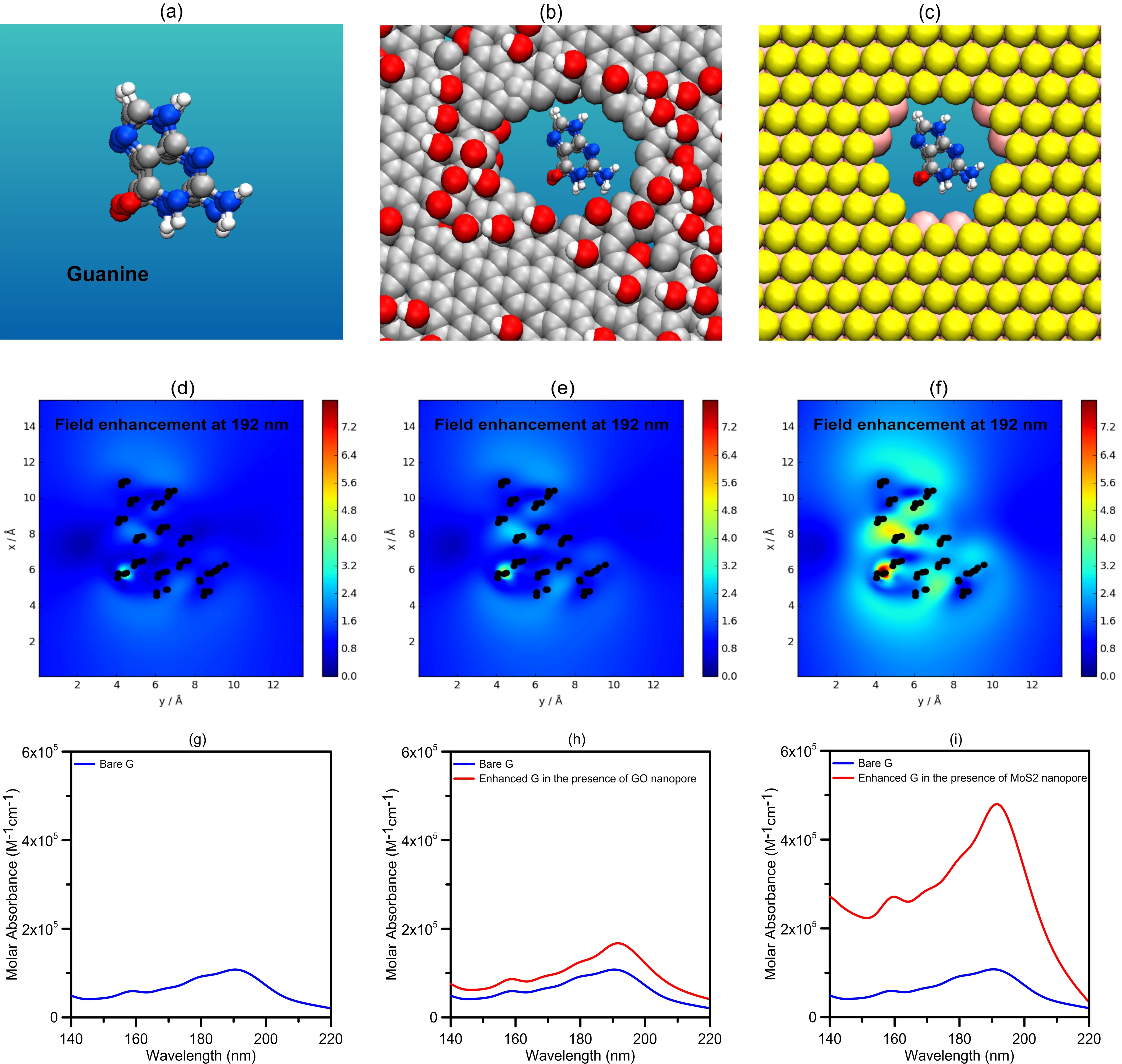** |
| --- |

**Figure S2.** The schematic structure of (a) bare G nucleobases, and in the presence of (b) GO and (c) MoS_2_ nanopores. The electric field enhancement of (d) bare G nucleobases, (e) at the GO and (f) MoS_2_ nanopore at 192 nm. The black points show the amplified G nucleobase atoms. At the peak wavelength of 192 nm (6.46 eV). The electric field of the G nucleobases at the GO and MoS_2_ nanopores is enhanced by a factor of 1.25 and 2.11, respectively. The molar absorbance of bare G nucleobases (g) and the enhanced absorbance of G nucleobases in the presence of (h) GO and (i) MoS_2_ nanopores. The enhancement factor of the G nucleobases absorbance at the presence of GO and MoS_2_ nanopores at the peak wavelength of 192 nm is about 1.56 and 4.48, respectively. The length of sheets is 5 nm.

| **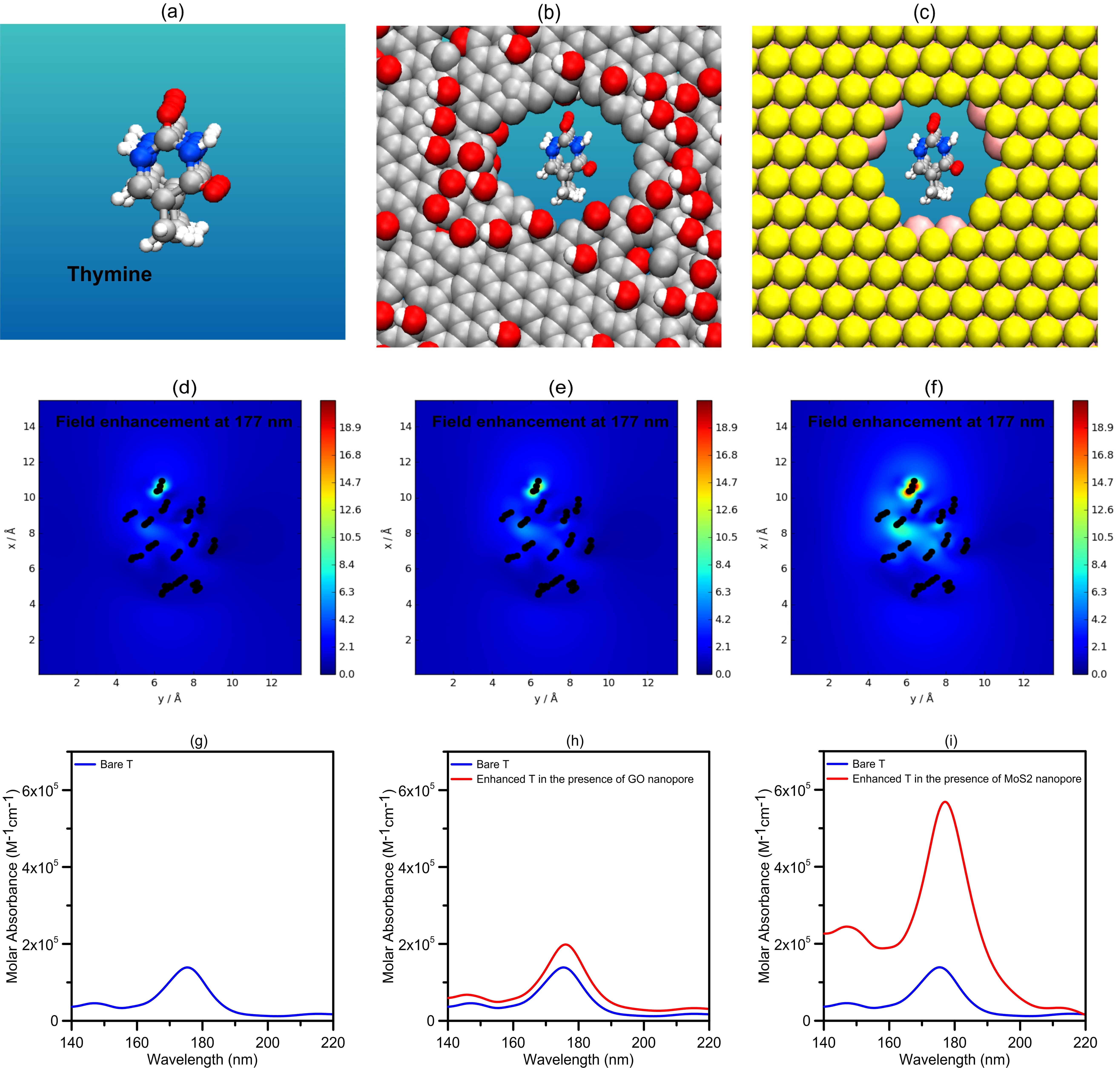** |
| --- |

**Figure S3.** The schematic structure of (a) bare T nucleobases, and in the presence of (b) GO and (c) MoS_2_ nanopores. The electric field enhancement of (d) bare T nucleobases, (e) at the GO and (f) MoS_2_ nanopore at 177 nm. The black points show the amplified T nucleobase atoms. At the peak wavelength of 177 nm (7 eV). The electric field of the T nucleobases at the GO and MoS_2_ nanopores is enhanced by a factor of 1.2 and 2.06, respectively. The molar absorbance of bare T nucleobases (g) and the enhanced absorbance of T nucleobases in the presence of (h) GO and (i) MoS_2_ nanopores. The enhancement factor of the T nucleobases absorbance at the presence of GO and MoS_2_ nanopores at the peak wavelength of 177 nm is about 1.44 and 4.27, respectively. The length of sheets is 5 nm.
